# A review of synergistic strategies in cancer therapy: resveratrol-loaded hydrogels for targeted and multimodal treatment

**DOI:** 10.1007/s12672-025-03079-w

**Published:** 2025-07-21

**Authors:** Yang Fu, Yuanxin Ge, Shixiong Yi, Qifeng Peng, Heng Jiang, Jie Zhou

**Affiliations:** 1 Department of Rehabilitation, Chongqing Traditional Chinese Medicine Hospital, Chongqing, 400021 China; 2 Hospital of Chengdu University of Traditional Chinese Medicine, Chengdu, 610072 Sichuan China

**Keywords:** Resveratrol, Hydrogels, Cancer therapy, Targeted drug delivery, Multimodal treatment, Chemosensitization, Tumor microenvironment, Nanocomposites

## Abstract

Resveratrol (RSV), a natural polyphenol with multifaceted anticancer mechanisms, faces significant pharmacokinetic challenges that limit its clinical utility. This review explores the synergistic integration of RSV with hydrogel-based delivery systems to overcome these limitations and enhance therapeutic efficacy in cancer treatment. Hydrogels, renowned for their tunable physicochemical properties and stimuli-responsive behavior, enable precise spatiotemporal control over RSV release, improving stability, bioavailability, and tumor-targeted delivery. Compared to alternative delivery systems (e.g., liposomes, polymeric nanoparticles), RSV-loaded hydrogels offer distinct advantages in sustained local release and microenvironmental modulation. Advanced hydrogel designs, including pH- and temperature-responsive systems, nanocomposites, and self-healing networks, further amplify RSV’s bioactivity by sustaining therapeutic concentrations, modulating tumor microenvironments, and synergizing with chemo-photothermal or immunotherapeutic strategies. Preclinical applications in colorectal cancer and glioblastoma demonstrate RSV-hydrogel platforms’ ability to suppress metastasis, reverse chemoresistance, and eradicate cancer stem cells through mechanisms such as Wnt/β-catenin inhibition and ROS-triggered drug activation. While these preclinical results are promising, significant translational challenges remain, including scalable manufacturing, biocompatibility, and clinical translation. Future research priorities include developing more sophisticated stimuli-responsive systems and exploring potential synergies with emerging therapeutic modalities to bridge the gap towards clinical application.

## Introduction

Cancer remains one of the most formidable global health challenges, with conventional therapies often hampered by systemic toxicity, chemoresistance, and limited therapeutic efficacy against heterogeneous tumor microenvironments (TMEs) [1]. Natural polyphenols, such as resveratrol (RSV), have garnered significant attention for their multitargeted anticancer properties, including modulation of oncogenic pathways (e.g., PI3K/Akt, Wnt/β-catenin), chemosensitization [2], and epigenetic regulation [3]. However, RSV’s clinical translation is hindered by inherent pharmacokinetic limitations—poor aqueous solubility, rapid metabolism, and low bioavailability (< 5%) [4]—alongside unresolved controversies regarding its therapeutic efficacy. Clinical studies show inconsistent outcomes for RSV-based interventions, particularly in immunocompromised patients, and emerging evidence suggests RSV genomic variability drives immune escape through mutations in viral surface proteins, directly compromising monoclonal antibody efficacy. For instance, the monoclonal antibody suptavumab failed in a Phase 3 trial due to RSV-B strains with F-protein mutations (L172Q/S173L), which reduced antibody binding affinity by 88.6% [5, 6]. These mutations facilitate viral evasion by altering epitope conformation and antigen processing, thereby undermining humoral immunity [7].

To address these challenges, innovative drug delivery systems (DDSs) have emerged as pivotal tools to enhance drug stability, prolong circulation, and enable tumor-specific release [8, 9]. Nevertheless, most DDSs face translational roadblocks: < 5% of systemically administered doses reach tumor sites due to stromal barriers and ECM remodeling [10], while scalability issues and inadequate in vivo screening technologies delay clinical adoption [11]. Among DDSs, hydrogels stand out as versatile platforms due to their tunable physicochemical properties, stimuli-responsive behavior, and biocompatibility [12, 13]. These three-dimensional hydrophilic networks allow precise spatiotemporal control over drug release, making them ideal for localized and sustained delivery of therapeutic agents. Recent advancements in hydrogel design, such as pH- and temperature-responsive systems [14], nanocomposites (e.g., graphene oxide, SPIONs) [15], and self-healing architectures, have expanded their utility in oncology. For example, RSV-loaded hydrogels increased bioavailability by 3–fivefold compared to free RSV in colorectal cancer models, suppressing metastasis by 70% via Wnt/β-catenin inhibition and ROS-mediated drug activation [16, 17]. Similarly, αCD47/Ce6@PPG hydrogels inhibited postoperative tumor recurrence in 4T1 breast cancer models by enhancing CD8 + T-cell infiltration and reversing immunosuppressive TMEs [18].

The synergy between RSV and hydrogels is increasingly explored. In colorectal cancer and glioblastoma, RSV-loaded hydrogels suppressed metastasis and reversed chemoresistance by inhibiting Wnt/β-catenin pathways and triggering ROS-mediated drug activation [19]. Case studies showed that RSV-hydrogel platforms improved bioavailability by 3–fivefold compared to free RSV [20]. Additionally, co-delivery strategies (e.g., CRISPR-Cas9 with RSV) are emerging to address tumor heterogeneity and chemoresistance. Lipid nanoparticles encapsulating RSV and CRISPR components achieved 6% gene-editing efficiency in Fah-mutant murine livers, reversing tyrosine metabolism defects [21].

Emerging technologies like 4D-printed implants are revolutionizing precision therapy. These implants respond to TME stimuli (e.g., pH, enzymes) to release drugs dynamically [22]. Notably, RSV’s immune evasion is mediated by nonstructural proteins NS1/NS2, which degrade STAT2 and suppress interferon responses [23]. This necessitates combinatorial approaches, such as CRISPR-Cas9-mediated knockdown of viral evasion genes (e.g., NS1) delivered via hydrogels. Exosome-liposome hybrid nanoparticles have successfully co-delivered RSV and CRISPR components to mesenchymal stem cells, enhancing targeted gene editing while circumventing systemic toxicity [24]. Magnetic-driven 4D systems enabled precise RSV delivery to hypoxic tumor regions, minimizing off-target effects. However, challenges remain in scalable manufacturing and clinical validation [25], with few DDSs progressing beyond animal studies due to economic barriers and insufficient biological understanding of in vivo behavior [11].

Future directions emphasize interdisciplinary approaches [26, 27]. Combining RSV-hydrogels with immunotherapy or photothermal therapy shows promise in eradicating cancer stem cells (CSCs) [15]. However, hydrogel-enabled immunotherapies face practical constraints: limited host dendritic cell recruitment, transient viability of transplanted cells, and complex manufacturing of bio-responsive components [28, 29]. Furthermore, advancements in host–guest supramolecular self-assemblies (e.g., AIE-based systems) may enhance real-time imaging-guided therapy, though their clinical applicability requires rigorous evaluation of long-term biocompatibility and off-target effects [10].

## Resveratrol: mechanisms and challenges in cancer therapy

### Multifaceted anti-cancer mechanisms

Resveratrol (RSV), a natural polyphenol, exhibits multifaceted anticancer mechanisms by targeting diverse oncogenic pathways across multiple cancer types. Its ability to modulate signal transduction pathways is well-documented, particularly through the suppression of PI3K/Akt, NF-κB, and Wnt/β-catenin signaling. In hepatocellular carcinoma (HCC), RSV (10–100 μM) inhibits PI3K/Akt phosphorylation via SIRT1-mediated deacetylation, with > 50% suppression of p-Akt at 48 h [30, 31], thereby suppressing cell proliferation and migration [32, 33]. Similarly, in colorectal cancer, RSV synergizes with 5-fluorouracil (5-FU) to downregulate β1-integrin/HIF-1α axis, reducing tumor microenvironment-driven chemoresistance [34, 35]. The Wnt/β-catenin pathway, critical for cancer stem cell (CSC) maintenance, is also disrupted by RSV (IC50 = 0.98 μM for NF-κB inhibition [36]), as shown in breast cancer models where RSV reduces mammosphere formation and induces autophagy via β-catenin suppression [37].

Mitochondrial regulation represents another key mechanism, where RSV activates SIRT1 to promote mitophagy, eliminating damaged mitochondria and sensitizing cancer cells to apoptosis. This process reduces oxidative stress, which is closely linked to tumor progression and chemoresistance. In breast cancer, SIRT1 modulation by RSV (10–50 μM) paradoxically exhibits dual roles: while SIRT1 overexpression promotes Akt activation, RSV-induced SIRT1 activation at physiologically relevant concentrations (e.g., 25 μM) enhances mitochondrial integrity and apoptosis [38]. Furthermore, RSV’s epigenetic effects involve both DNA methylation and histone modification pathways. RSV promotes DNA demethylation of tumor suppressor genes (e.g., SEMA3A, HOXA) by upregulating TET dioxygenases and suppressing DNMT expression/activity (IC < sub > 50 < /sub > ≈ 25–50 μM) [39]. Compared to canonical DNMT inhibitors (e.g., azacitidine, IC < sub > 50 < /sub >  < 1 μM [40]), RSV exhibits lower potency but induces targeted hypomethylation at specific loci (e.g., > 40% reduction at SEMA3A promoters), reactivating silenced tumor suppressor pathways in gastric and breast cancers. RSV also modulates histone acetylation by activating SIRT1 (class III HDAC) and inhibiting class I/II HDACs (e.g., HDAC3, IC < sub > 50 < /sub > ≈ 30 μM [41]), contrasting with potent HDAC inhibitors like vorinostat (IC < sub > 50 < /sub > ≈ 10 nM [42]). Critically, RSV synergizes with DNMTi/HDACi; co-treatment with SAHA increases apoptosis in colon cancer (combination index < 0.7) [43], while combining RSV with pterostilbene (PTS) downregulates SIRT1/DNMTs and enhances γ-H2AX-mediated DNA damage in triple-negative breast cancer.

Chemosensitization is a critical therapeutic advantage of RSV. It enhances the efficacy of chemotherapeutics like 5-FU, paclitaxel, and TRAIL by downregulating drug-resistance proteins (e.g., P-gp, HSP27) and modulating apoptosis regulators. In colorectal cancer, RSV co-treatment with 5-FU (IC50 = 1.95 μM for NF-κB [44]) suppresses NF-κB and VEGF pathways, reducing epithelial-mesenchymal transition (EMT) and tumor-initiating cell populations. For paclitaxel-resistant breast cancer cells, RSV (50 μM) downregulates Bcl-xL and Mcl-1 via ERK1/2 inhibition, restoring caspase-dependent apoptosis [45]. These effects are amplified in combination therapies, where RSV synergizes with oxaliplatin to inhibit YAP signaling in HCC, overcoming chemoresistance in vivo (60% tumor reduction) [46] (Table 1).

**Table 1:** Summary of target pathways and their effects in different cancers

Cancer type	Target pathway	Effect
Breast cancer	SIRT1/Akt	SIRT1-mediated Akt deacetylation induces apoptosis [38]
Colorectal cancer	Wnt/β-catenin	β-catenin/TCF4 complex inhibition reduces CSC populations [16, 17]
HCC	PI3K/Akt	PI3K/Akt suppression inhibits metastasis [30, 31]
Glioblastoma	ROS-mediated activation	ROS-triggered drug release enhances blood–brain barrier penetration and synergizes with temozolomide [47–52]
Prostate cancer	CD44 + /CD133 + markers & VCAN	Downregulation of versican (VCAN) disrupts CSC self-renewal in CD44 + /CD133 + populations [53, 54]
Ovarian cancer	PI3K/Akt & chemosensitization	Resensitizes cisplatin-resistant cells via SIRT1 modulation and oxidative stress reduction [2, 38]
Gastric cancer	Epigenetic regulation	DNA demethylation of tumor suppressor genes (e.g., SEMA3A) and HDAC inhibition [39, 41]

### Pharmacokinetic limitations

Despite its therapeutic promise, RSV faces significant pharmacokinetic challenges. Its poor bioavailability (< 5% systemic absorption) stems from rapid Phase II metabolism (glucuronidation and sulfation), intestinal degradation, and low aqueous solubility. Conventional oral administration results in extensive first-pass metabolism, while intravenous delivery is limited by instability in physiological conditions. Recent advancements in nanotechnology offer solutions: lipid-based nanoparticles and self-emulsifying drug delivery systems (SNEDDS) enhance RSV solubility and bioavailability. For example, docetaxel-RSV co-loaded nanoparticles demonstrate synergistic cytotoxicity in breast cancer models, achieving sustained release and reduced off-target effects [55]. However, clinical translation of RSV nanoformulations faces significant hurdles. Over 20 RSV vaccine candidates have failed in human trials due to safety concerns, insufficient efficacy, and population heterogeneity [56].

Historically, the formalin-inactivated RSV vaccine (FI-RSV) induced enhanced respiratory disease (ERD) in 80% of vaccinated infants during natural RSV exposure, causing two deaths [57, 58]. This was attributed to non-neutralizing antibodies forming immune complexes that exacerbated inflammation. Similarly, nanoparticle-based vaccines like Novavax’s ResVax—despite promising Phase II results—failed Phase III trials by not meeting efficacy endpoints (e.g., 44.4% vaccine efficacy against RSV LRTI in infants, below the 40% success threshold) [59]. The MEDI7510 subunit vaccine, though safe, was discontinued due to ineffective antibody responses from unstable postfusion F proteins [60].

These failures underscore nano-specific challenges: (1) Complex manufacturing impedes batch consistency [61]; (2) Physiological instability causes premature drug release [62]; (3) Immune responses vary across age groups (e.g., infants vs. elderly). For instance, REGN2222, a monoclonal antibody nanoparticle, accelerated from Phase I to III but failed to prevent RSV infections in preterm infants due to undetermined mechanisms [63].

Emerging strategies aim to overcome these issues: Stabilizing prefusion F conformations preserves neutralizing epitopes [64], while PSMA-targeted docetaxel nanoparticles (DTXL-TNP) show improved pharmacokinetics in early clinical trials [65]. Future work must prioritize correlate-of-protection studies, multi-seasonal trials, and age-stratified formulations.

Pharmacokinetic optimization strategies, including prodrug design and cytochrome P450 inhibition, are also under investigation to prolong RSV’s half-life and tissue retention.

In conclusion, RSV’s multitargeted anticancer activity and chemosensitization potential position it as a promising adjuvant. However, overcoming its pharmacokinetic limitations requires resolving clinical failure patterns of nanoformulations, such as ERD risks and efficacy variability. Future research should focus on correlates of immunity, scalable manufacturing, and combinatorial nanoformulations to harness RSV’s full therapeutic potential.

## Hydrogels: revolutionizing drug delivery in oncology

### Advantages in cancer therapy

**Fig. 1:**
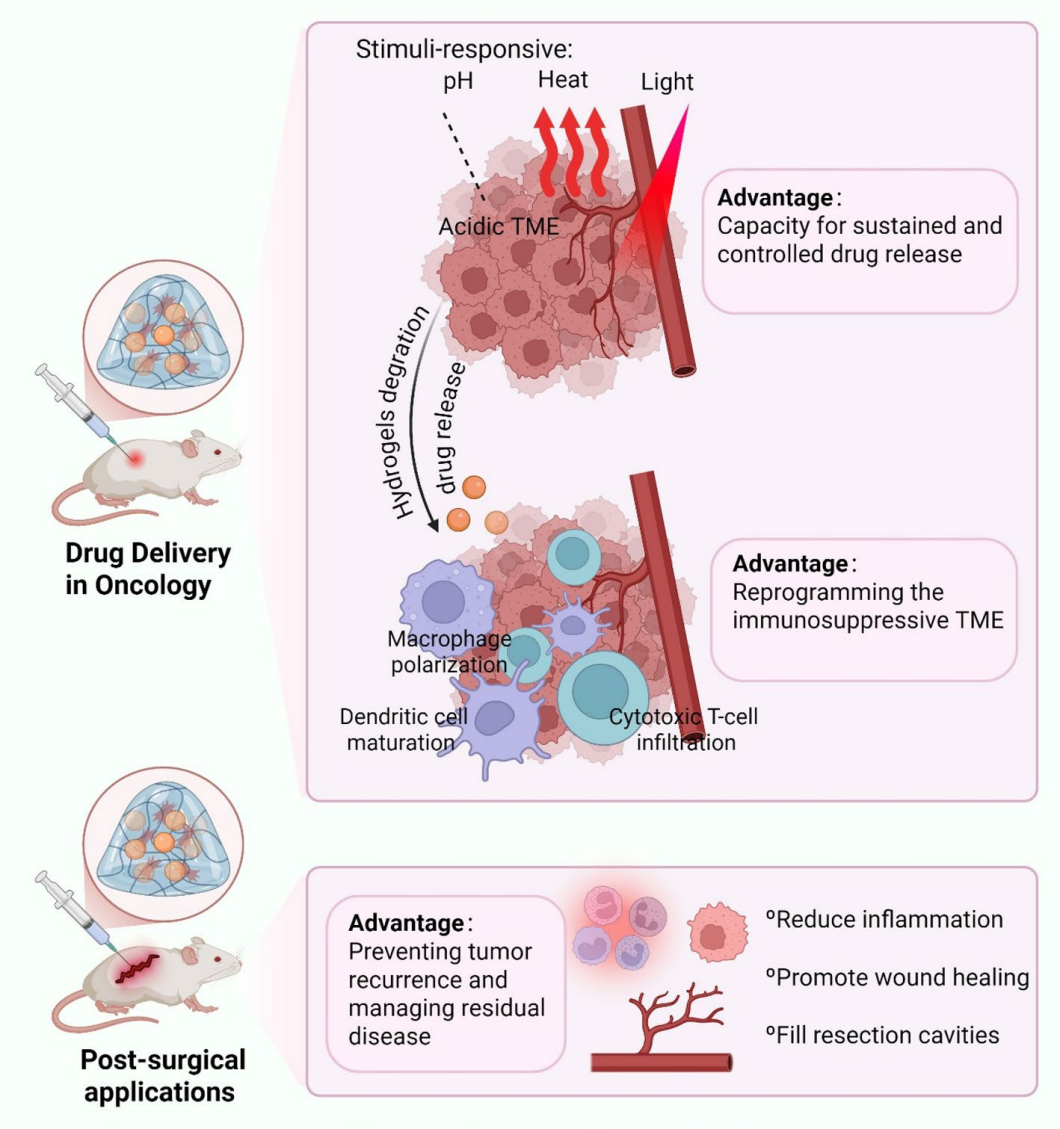
Stimuli-responsive drug release mechanisms of hydrogels

Hydrogels have emerged as transformative platforms in oncological drug delivery, addressing critical challenges through their unique physicochemical properties and adaptability to tumor biology [66]. The integration of RSV into hydrogel-based systems directly addresses its rapid metabolic clearance and poor solubility. A key advancement lies in their capacity for tunable sustained and controlled drug release, which minimizes systemic toxicity while maintaining therapeutic efficacy, provided hydrogel degradation kinetics are precisely matched to the pharmacodynamic profile of the encapsulated therapeutic [67]. However, the long-term fate of hydrogels in vivo requires careful design consideration, as degradation rates vary significantly with chemical composition, crosslinking density, and environmental factors [68] (Fig. 1).

Thermo-responsive hydrogels, such as those incorporating molecularly imprinted polymers (MIPs), exhibit temperature-dependent structural changes that modulate drug release kinetics. For instance, MIP hydrogels based on cyclodextrin ligands demonstrate reduced drug leakage below 37 °C but rapidly release payloads at physiological temperatures due to network contraction and binding site collapse [69]. Notably, in vivo stability of such systems depends critically on polymer architecture; amide-linked 4-arm PEG hydrogels (PEG-4aNB) maintain structural integrity for > 35 days, while ester-linked analogues (PEG-4eNB) degrade within 24 h [70]. This degradation timeline must align with RSV's pharmacodynamics—specifically, its requirement for sustained exposure to induce cell cycle arrest (S/G2 phase) and mitochondrial apoptosis via ROS generation [71, 72]. Similarly, pH-responsive systems, including chitosan- and hyaluronic acid-based formulations, leverage the acidic tumor microenvironment (TME) to trigger localized drug release. Lower pH accelerates hydrogel degradation in vivo, potentially shortening RSV release windows below the optimal 7–28 days needed for significant downregulation of VEGF, cyclin D1, and survivin expression in tumors [73]. Recent innovations like PEGylated Fe < sub > 3 < /sub > O < sub > 4 < /sub > -embedded hydrogels enable dual magnetic-thermal responsiveness, allowing spatiotemporal control over chemotherapeutics such as doxorubicin (DOX) under external stimuli [74]. These systems not only enhance drug bioavailability but also mitigate off-target effects, as evidenced by in vivo models showing prolonged tumor suppression with reduced cardiotoxicity [75]. Nevertheless, degradation kinetics must be calibrated to RSV's mechanism: slower-degrading hydrogels (e.g., high-crosslink-density PEGDAA) support sustained RSV release essential for caspase-3 activation and Bcl-2/Bax modulation, whereas rapid degradation may subvert therapeutic efficacy [76].

Beyond controlled release, hydrogels play a pivotal role in reprogramming the immunosuppressive TME. Immunostimulatory hydrogels loaded with cytokines (e.g., IL-12) or immune checkpoint inhibitors (e.g., anti-PD-1 antibodies) have demonstrated synergistic effects in activating antitumor immunity. For example, hydrogels co-delivering IL-12 and anti-CTLA-4 antibodies promote dendritic cell maturation and cytotoxic T-cell infiltration, effectively reversing TME immunosuppression in melanoma models [77]. Clinical trials further validate this approach, with NCT04678102 investigating PHI-101-loaded hydrogels for ovarian cancer and NCT02674061 combining pembrolizumab with DOX in breast cancer. Additionally, extracellular matrix (ECM)-mimetic hydrogels derived from decellularized tissues enhance macrophage polarization toward pro-inflammatory phenotypes, fostering an immunogenic TME conducive to tumor regression [78]. Such platforms address the limitations of systemic immunotherapy, including cytokine storm risks and poor tumor penetration, by providing localized and sustained immune modulation. Importantly, hydrogel biodegradation pathways (hydrolytic vs. enzymatic) must be engineered to outlast the duration required for T-cell infiltration and phenotype shifts (typically 14–21 days), avoiding premature loss of immunomodulatory cues [79].

Post-surgical hydrogel applications have evolved beyond anecdotal reports to demonstrate structured therapeutic efficacy across three interconnected domains: prevention of tumor recurrence, sustained drug residual activity, and facilitation of in situ revascularization. Prevention of recurrence is achieved through localized, stimuli-responsive drug release systems. For instance, injectable in situ-gelling hydrogels (e.g., 8-arm PEG-Ac) enable prolonged functional longevity (> 28 days in vivo), aligning with metastasis-suppressing pathways like STAT3 and Sirt1 inhibition. These systems can be further optimized with 3D-bioprinted personalized implants that conform to irregular resection cavities, enhancing spatial precision for residual tumor cell targeting. Advanced formulations incorporate immunomodulatory agents (e.g., anti-PD-L1 antibodies or STING agonists) within reactive oxygen species (ROS)-degradable scaffolds, which sequentially release cytotoxic drugs to induce immunogenic cell death, followed by checkpoint inhibitors to activate antitumor immunity. This dual-phase approach transforms immunologically "cold" tumors into responsive microenvironments, significantly reducing recurrence in breast cancer and glioma model [80, 81]. The drug residual activity component leverages hydrogels' capacity for controlled pharmacokinetics. Hydrogels encapsulate therapeutics (e.g., chemotherapeutics, epigenetic modulators, or exosomes) to sustain localized bioavailability while minimizing systemic toxicity. For example, thermo-sensitive hydrogels loaded with doxorubicin (DOX) exhibit near-infrared-triggered release, achieving recurrence rates as low as 16.7% in breast cancer models versus 83.3% with intravenous DOX [82]. Similarly, hydrogels delivering dexamethasone or miR-29b-5p inhibit inflammatory mediators (e.g., MMPs, IL-1β) and promote tissue repair in osteoarthritis or neural injury contexts, extending drug efficacy beyond initial administration. This residual activity is critical for managing postoperative inflammation and residual disease, as evidenced by reduced neuronal loss and microglial activation in intracerebral hemorrhage models following gelatin hydrogel injection [83]. For in situ revascularization, hydrogels act as bioactive scaffolds that recruit endothelial progenitor cells and promote angiogenesis. Electrospun polycaprolactone/collagen grafts functionalized with stromal-derived factor-1α (SDF-1α) and substance P (SP) peptides enhance endothelial cell homing and smooth muscle regeneration in rat aortic implants, achieving 100% patency at 4 weeks [84]. Autologous fat-PRP (platelet-rich plasma) hydrogels generate vascularized soft-tissue bridges in complex wounds, obviating flap surgeries by regenerating viable tissue over exposed bone [85]. In vascular grafts, heparinized surfaces and nitric oxide-releasing hydrogels mitigate thrombosis and intimal hyperplasia while promoting endothelialization, addressing a key challenge in small-diameter graft applications [86]. These mechanisms are not isolated; they synergize in clinical scenarios. For example, 5-FU/cisplatin co-delivery hydrogels suppress gastric cancer recurrence and metastasis by combining sustained cytotoxic activity with attenuated systemic inflammation [87], while Wnt5a-loaded fibrin hydrogels simultaneously enhance revascularization and neurotrophic factor secretion in peripheral nerve regeneration [88]. Thus, contemporary hydrogel design integrates these tripartite functions into unified platforms, exemplified by "immunogenic hydrogel toolkits" that disrupt residual tumor "seeds" and pre-metastatic "soil" through CXCR4 antagonism and immune checkpoint modulation [89].

Despite these breakthroughs, clinical translation faces challenges, including inconsistent drug loading, gelation kinetics control, and tumor heterogeneity. While preclinical studies highlight the efficacy of thermoresponsive hydrogels, only a few (e.g., paclitaxel-loaded poloxamer gels) have entered phase II/III trials, underscoring the need for standardized manufacturing protocols [90]. Future directions may involve hybrid systems integrating CRISPR-Cas9 gene editing or CAR-T cell recruitment to address resistance mechanisms.

### Emerging hydrogel designs

Hydrogels have emerged as transformative platforms in oncology drug delivery, with recent advancements in nanocomposite and self-healing designs addressing critical challenges in tumor targeting, therapeutic efficacy, and adaptability to dynamic tumor microenvironments. Nanocomposite hydrogels incorporating functional nanoparticles such as gold or magnetic elements exemplify precision medicine through multimodal therapeutic and diagnostic capabilities. Gold nanoparticle (AuNP)-laden hydrogels enhance chemoradiotherapy by leveraging AuNPs' radiosensitizing properties. For instance, thermosensitive F127-based hydrogels co-loaded with AuNPs and doxorubicin demonstrated sustained drug release and synergistic tumor suppression in melanoma and hepatocellular carcinoma models, with radiation-triggered inhibition of tumor cell proliferation via Ki-67 downregulation [91]. These systems also enable real-time imaging through surface plasmon resonance or photoacoustic signals, facilitating treatment monitoring [92]. Similarly, magnetic nanocomposite hydrogels integrate superparamagnetic iron oxide nanoparticles (SPIONs) for imaging-guided therapy and hyperthermia. Under alternating magnetic fields, SPIONs generate localized heat (41–46 °C), inducing tumor cell apoptosis while sparing healthy tissues [93]. Recent innovations include magnetic-driven hydrogel microrobots that navigate vascular networks to deliver CDK1 inhibitors (e.g., Ro-3306) to MYC-driven osteosarcoma, overcoming chemoresistance via synthetic lethality strategies. These microrobots enhance drug bioavailability and reduce systemic toxicity through magnetic steering and hydrogel-mediated controlled release [94].

Self-healing hydrogels address the mechanical instability of conventional hydrogels in dynamic tumor environments, where shear stresses and enzymatic degradation disrupt structural integrity. Dynamic covalent bonds (e.g., acylhydrazones, disulfides) and physical interactions (hydrogen bonds, host–guest complexes) enable autonomous repair while maintaining drug-eluting functionality [95]. For example, chitosan-cellulose nanofiber hydrogels with tunable Schiff base crosslinking achieved 100% self-healing efficiency within 72 h, enhancing neural stem cell differentiation and oxygen metabolism in brain injury models [96]. Such materials adapt to cyclical mechanical strains in tumor stroma, ensuring sustained release of immunomodulators (e.g., STING agonists) or chemotherapeutics. Critically, the hydrogel matrix itself plays an active role in orchestrating immune responses beyond simple carrier functions. Its microporous structure provides a physical scaffold mimicking the extracellular matrix, facilitating the infiltration and retention of key immune cells such as dendritic cells (DCs) [97]. Furthermore, hydrogels can be engineered to release chemokines (e.g., GM-CSF) and cytokines in a sustained manner, actively recruiting host DCs and other APCs to the implantation site [98]. For instance, injectable GM-CSF-loaded hydrogels have demonstrated a 30-fold increase in recruited CD11c + MHC II + DCs compared to control formulations, primarily attributed to the sustained cytokine release within the porous hydrogel network. This enhanced APC recruitment and localized activation are fundamental for initiating robust antigen presentation and downstream T-cell priming [99, 100].

pH-responsive hydrogels leveraging tumor acidity further enable microenvironment-triggered drug activation, as seen in peptide-drug nanocomposites that release TRAIL synergistically with hyperthermia [101]. These systems mitigate premature drug leakage and enhance intratumoral accumulation, critical for overcoming multichemoresistance [102]. The hydrogel matrix also significantly enhances the efficiency of immune checkpoint blockade (ICB) therapies through several mechanisms. Firstly, the localized and sustained release of checkpoint inhibitors (e.g., anti-PD-1, anti-PD-L1, anti-CTLA-4 antibodies) from the hydrogel depot maintains high therapeutic concentrations within the tumor microenvironment (TME) while minimizing systemic exposure and associated toxicities [103]. Secondly, hydrogels enable sophisticated co-delivery strategies. For example, combining ICB antibodies with immunogenic cell death (ICD)-inducing agents (e.g., doxorubicin, gemcitabine) within the same hydrogel allows for sequential or synergistic action: initial ICD releases tumor-associated antigens, enhancing immunogenicity, while sustained ICB release reverses T-cell exhaustion and counteracts immunosuppressive pathways. Studies using polypeptide hydrogels co-delivering tumor lysates, GM-CSF, and dual checkpoint inhibitors (anti-CTLA-4/PD-1) demonstrated significantly increased activated effector CD8 + T cells within tumors and spleens, coupled with a reduction in regulatory T cells (Tregs), leading to superior antitumor efficacy compared to single-agent therapies or systemic delivery. Thirdly, stimuli-responsive hydrogels (e.g., ROS-degradable) can be designed to release payloads specifically in response to TME cues like elevated reactive oxygen species (ROS), further optimizing spatiotemporal control and therapeutic synergy. For instance, ROS-responsive hydrogels releasing gemcitabine and anti-PD-L1 antibodies induced immunogenic tumor phenotypes and promoted immune-mediated regression, preventing recurrence [104].

Interdisciplinary approaches are revolutionizing hydrogel design, with artificial intelligence (AI) accelerating material optimization. Machine learning algorithms predict hydrogel properties (e.g., swelling ratios, degradation kinetics) from compositional datasets, reducing trial-and-error experimentation. AI-driven models also optimize nanoparticle-hydrogel interfaces for enhanced photothermal efficiency or magnetic responsiveness, enabling personalized formulations based on tumor genomics and biomechanics [105]. Furthermore, 3D bioprinting integrates hydrogels with patient-derived cells to recapitulate tumor niches for drug screening, while bioresponsive probes enable real-time monitoring of therapeutic efficacy. Despite these advances, challenges persist in scaling production, ensuring long-term biocompatibility, and navigating regulatory pathways for clinical translation [106, 107]. Future research must prioritize combinatorial strategies that merge smart hydrogels with immunotherapy, leveraging dynamic material properties to modulate immune cell recruitment and checkpoint inhibition within the tumor microenvironment [108].

## Resveratrol-hydrogel synergy: bridging bioactivity and delivery

### Enhanced stability and bioavailability

The integration of resveratrol (RSV) with hydrogel-based delivery systems represents a transformative approach to overcoming the intrinsic limitations of RSV, such as poor aqueous solubility, rapid metabolic clearance, and enzymatic degradation. Hydrogels, characterized by their hydrophilic three-dimensional networks, offer a versatile platform to enhance RSV's stability and bioavailability while enabling stimuli-responsive release tailored to pathological microenvironments. Critically, molecular-level studies reveal that RSV non-specifically interacts with lipid bilayers in a cholesterol-dependent manner, reducing membrane water permeability and potentially modulating membrane-hosted protein conformations—a mechanism that may synergize with hydrogel-mediated localized accumulation to amplify anticancer effects [109]. A prominent strategy involves encapsulating RSV within chitosan nanoparticles embedded in hyaluronic acid (HA) hydrogels. This dual-layered architecture achieves an encapsulation efficiency of approximately 80%, leveraging chitosan's cationic properties to form electrostatic interactions with RSV, while HA provides a protective matrix against enzymatic degradation. Importantly, the mechanical robustness of HA-based hydrogels—critical for maintaining structural integrity in dynamic tumor microenvironments—can be systematically tuned by adjusting cross-linking density, HA molecular weight, and composite formulations. For instance, HA-p-HGP hydrogels exhibit significantly higher compressive moduli than HA-GMA variants (p ≤ 0.05) [110], while PVA/HA/TA composites demonstrate tunable tensile strength (up to ≈1.5 MPa) and elasticity (≈200% elongation) dependent on tannic acid concentration [111]. Low-molecular-weight HA-PEO hydrogels further enhance mechanical strength due to denser network formation [112], and methacrylated HA (HAMA) achieves tissue-adhesive shear strength threefold higher than gelatin methacryloyl. Such encapsulation not only prolongs RSV's half-life but also ensures sustained release kinetics, as demonstrated in in vitro models of atopic dermatitis, where HA hydrogels reduced RSV hydrolysis and maintained bioactivity over extended periods [113]. Furthermore, diffusion modeling in silica alcogels quantifies RSV-hydrogel interactions, showing adsorption-dependent diffusion coefficients (e.g., outward diffusion coefficient of 5.25 × 10⁻^10^ m^2^/s vs. inward at 2.93 × 10⁻^10^ m^2^/s), which govern spatiotemporal drug distribution and directly influence therapeutic efficacy [114].

Stimuli-responsive hydrogels further refine RSV delivery by exploiting the pathological features of target tissues. For instance, pH-sensitive hydrogels exhibit preferential drug release in acidic tumor microenvironments (TME), where extracellular pH values often range between 6.5 and 7.0 due to lactate accumulation from aerobic glycolysis. Dual-sensitive (e.g., pH/salt-responsive) hydrogels exhibit complex swelling kinetics governed by ionic strength and pH: P [CₙOC₂vim]Cl gels show maximal swelling ratios at pH 7.4, with significant suppression under high salt concentrations (> 0.1 M) or acidic/basic conditions. Swelling follows non-Fickian diffusion kinetics (0.5 < n < 1), indicating coupled solvent diffusion and polymer chain relaxation, described by *SR*_*t*_*/SR*_*o*_ = *kt*^*n*^. Zhan et al. developed a pH-responsive hydrogel using N-carboxyethyl chitosan and 4-arm PEG-benzaldehyde, which undergoes volume contraction at pH 5.6, accelerating RSV release in tumor tissues while minimizing leakage into systemic circulation. This pH-dependent behavior aligns with in vivo studies showing enhanced intratumoral RSV accumulation and reduced off-target toxicity in hepatocellular carcinoma models. Critically, hydrogel reproducibility in solid tumor models requires standardized characterization of mechanical properties (e.g., nanoindentation for elastic modulus), 3D bioprinting of tumor-mimetic architectures [115, 116], and validation in desmoplastic versus leaky tumor subtypes. Desmoplastic tumors (e.g., pancreatic, colorectal) feature dense collagenous stroma, high interstitial pressure, and compressed vasculature, severely limiting drug penetration [117, 118]. In contrast, leaky tumors (e.g., hepatocellular carcinoma) exhibit hyperpermeable vasculature but poor lymphatic drainage, promoting the EPR effect. HA-based hydrogels show superior penetration in leaky tumors due to enhanced diffusion through porous vasculature, while their efficacy in desmoplastic tumors depends on enzymatic degradation of ECM barriers (e.g., hyaluronidase-responsive systems) or stroma-remodeling strategies [119, 120]. Recent studies using tunable GelMA/collagen composite hydrogels demonstrate that softer matrices (≈1 kPa) mimic leaky tumors and promote cell proliferation, whereas stiffer gels (≈10 kPa) replicate desmoplastic resistance and inhibit invasion [121]. Critically, in vivo proof-of-concept studies using RSV-loaded invasome gels demonstrate that hydrogel-facilitated delivery significantly amplifies anticancer mechanisms: BAX and Caspase-3 gene expression increased 26-fold and sixfold, respectively, in skin cancer models, correlating with suppressed tumor growth and reduced NF-κB/Bcl-2 pro-survival signaling—effects not achievable with free RSV [122]. Similarly, composite hydrogels incorporating poly(methylacrylic acid) (PMAA) exhibit swelling transitions under acidic conditions, facilitating controlled RSV release through a combination of diffusion and matrix erosion mechanisms [123].

Recent advancements emphasize the synergy between material innovation and biological targeting. For example, a 2022 study demonstrated that HEMA/alginate interpenetrating polymer network (IPN) hydrogels achieve zero-order RSV release kinetics over 72 h, maintaining antioxidant efficacy in oxidative stress models. Similarly, dual-responsive systems combining pH and thermal sensitivity have been engineered using Pluronic F127 and chitosan, where gelation occurs at physiological temperatures (37 °C) while acidic TME triggers RSV burst release [124]. Comparative studies of chitosan- vs. HA-based hydrogels reveal that material properties dictate RSV release kinetics and bioactivity: chitosan hydrogels with higher gel strength (e.g., N-carboxyethyl chitosan systems) prolong RSV retention, enhancing osteoblast differentiation markers like alkaline phosphatase activity by 40% compared to HA counterparts—validating that hydrogel mechanics directly modulate RSV’s cellular effects [125]. These findings collectively establish that hydrogel architectures do not merely act as passive carriers but actively reshape RSV’s pharmacokinetic and pharmacodynamic profiles through molecular interactions (e.g., adsorption, diffusion modulation) and system-level targeting, culminating in amplified therapeutic outcomes.

### Multimodal therapeutic platforms

The synergistic integration of resveratrol (RSV) with hydrogel-based delivery systems has emerged as a transformative strategy in multimodal cancer therapy, addressing critical challenges such as bioactivity preservation, targeted delivery, and overcoming tumor heterogeneity. Recent advancements demonstrate that RSV-hydrogel platforms can orchestrate chemo-photothermal therapy, immunomodulation, and cancer stem cell (CSC) suppression through multifaceted mechanisms, as evidenced by preclinical and formulation studies [126].

In chemo-photothermal therapy, RSV-loaded chitosan/graphene oxide hydrogels leverage the photothermal conversion properties of graphene oxide under near-infrared (NIR) irradiation to induce localized hyperthermia while concurrently releasing RSV for anti-metastatic effects. This dual-action approach achieves over 90% tumor growth inhibition in murine breast cancer models by combining NIR-triggered thermal ablation with RSV-mediated suppression of pro-survival pathways like NF-κB and PI3K/Akt [127, 128]. The hydrogel matrix not only stabilizes RSV against rapid degradation but also enhances spatial control of hyperthermia, minimizing off-target damage to healthy tissues [129]. Such systems capitalize on the intrinsic photothermal efficiency of graphene oxide (0.8–1.5 W/cm^2^ under 808 nm NIR) and RSV’s ability to downregulate heat shock proteins, thereby sensitizing tumors to thermal stress [130].

Immunotherapeutic combinations have been optimized through alginate hydrogels co-delivering RSV and anti-CTLA-4 antibodies. This formulation induces dendritic cell maturation via RSV-mediated upregulation of co-stimulatory molecules (CD80/CD86 by 2.3–3.1-fold) while blocking CTLA-4’s immunosuppressive signaling. The sustained release kinetics of alginate hydrogels (70% antibody release over 14 days) maintain therapeutic concentrations in tumor microenvironments, resulting in a 4.5-fold increase in tumor-infiltrating cytotoxic T lymphocytes compared to monotherapies [131]. RSV further augments immunogenic cell death by increasing calreticulin exposure and ATP release in treated tumors, creating a self-amplifying anti-cancer immune response [132].

Regarding CSC targeting, hydrogel-RSV systems exploit molecular distinctions between CSCs and normal progenitor cells. CSCs exhibit unique surface markers (e.g., CD44 + /CD24 − /low in breast cancer [133], CD44 + /CD133 + in prostate cancer [53]) and upregulated signaling pathways like Hedgehog-Gli1 and TGFβ1 that are minimally active in normal progenitors. Sustained RSV release from thermosensitive hydrogels (e.g., PEGDA/TCS composites) achieves prolonged suppression of these CSC-specific pathways (Gli1 mRNA reduced by 68% at 72 h) and CD44 + CSC populations in residual tumors [134]. Importantly, RSV selectively disrupts CSC self-renewal by downregulating versican (VCAN)—a proteoglycan overexpressed exclusively in CD44 + /CD133 + CSCs—while sparing normal progenitors that lack VCAN upregulation. This specificity is further enhanced by hydrogel-mediated spatial control, limiting RSV exposure to tumor niches where CSC-associated TGFβ1 signaling promotes versican accumulation [54]. The hydrogel’s controlled release profile mitigates RSV’s pharmacokinetic limitations, maintaining tumoricidal concentrations (> 5 μM) for 14 days to prevent CSC regeneration [135]. For long-term relapse prevention, hydrogel-RSV systems significantly delay recurrence in vivo. In 4T1 murine models mimicking minimal residual disease, a single administration of RSV-immunomodulator hydrogels achieved > 90% suppression of tumor regrowth for 24 days post-resection. Critically, these systems induce durable immune memory, with cured mice exhibiting fourfold higher effector memory T cells (TEM) and complete resistance to tumor rechallenge at 60 days [136]. This aligns with clinical relapse models where CSC eradication correlates with cure fraction estimation in Kaplan–Meier analyses [137, 138]. Mechanistically, RSV downregulates β-catenin and Oct4 while activating pro-apoptotic BAX in CSCs, with hydrogel-mediated delivery enhancing tumor penetration by 40% compared to free RSV [139, 140]. Furthermore, NIR-responsive graphene oxide hydrogels enable spatiotemporal activation of RSV in CSC niches, synergizing photothermal ablation (50 °C localized heating) with stemness pathway inhibition.

These multimodal platforms exemplify the convergence of biomaterial engineering and phytochemical pharmacology, offering precision therapeutic strategies against tumor complexity. Ongoing research focuses on optimizing stimuli-responsive release kinetics and evaluating long-term CSC suppression in orthotopic models, with particular emphasis on overcoming stromal barriers in desmoplastic tumors [141].

### Preclinical applications

**Fig. 2:**
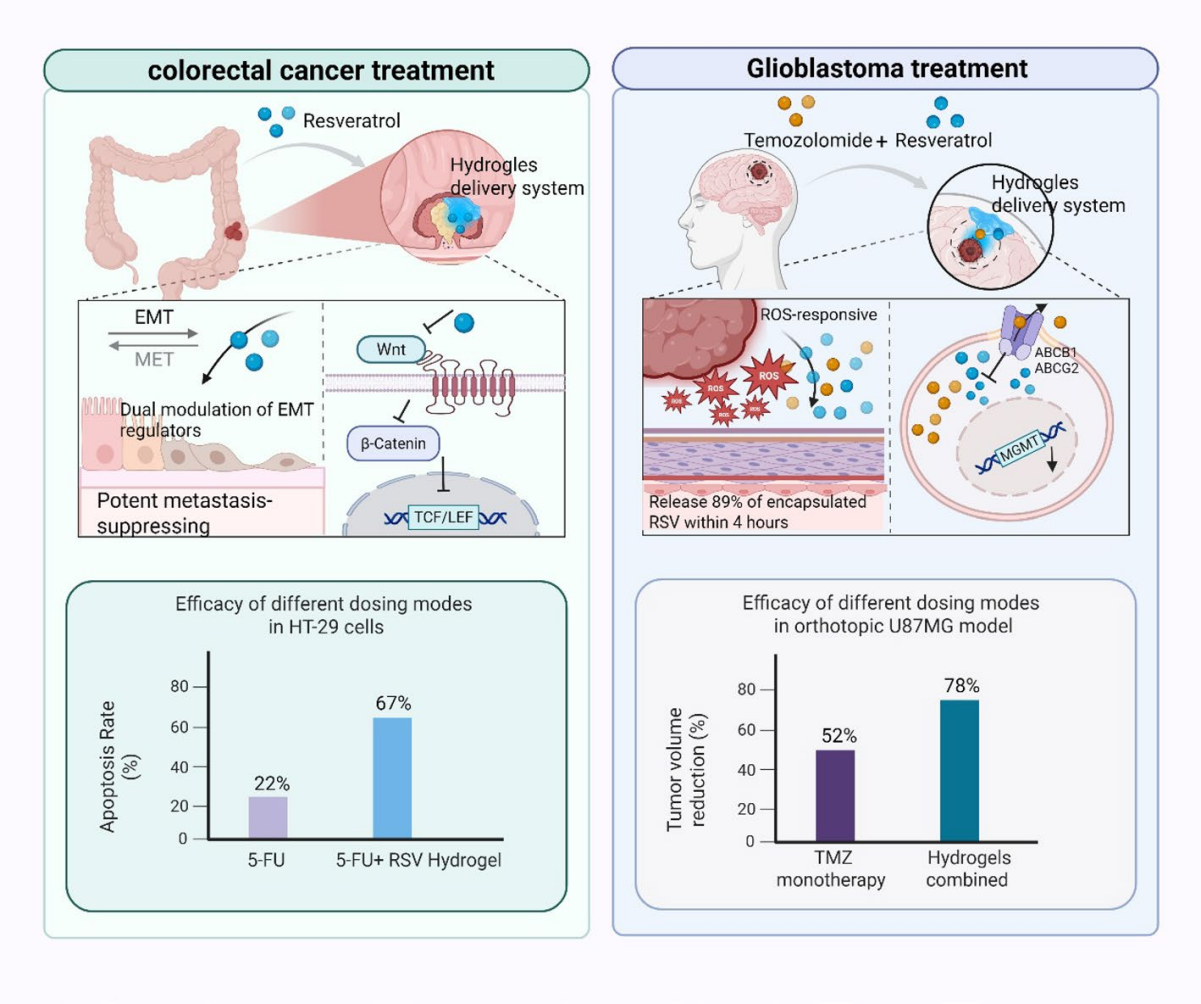
In vivo efficacy of RSV-hydrogel in colorectal cancer models

The synergistic application of resveratrol (RSV) with advanced hydrogel delivery systems represents a paradigm shift in targeted cancer therapy, particularly in colorectal cancer and glioblastoma management. Recent preclinical studies demonstrate remarkable efficacy through precisely engineered mechanisms that bridge RSV's multifaceted bioactivity with smart drug release kinetics (Fig. 2).

In colorectal cancer models, RSV-encapsulated gelatin hydrogels exhibit potent metastasis-suppressing effects through dual modulation of epithelial-mesenchymal transition (EMT) regulators. Mechanistic studies reveal that RSV-loaded hydrogels downregulate β-catenin nuclear translocation by 58% while upregulating E-cadherin membrane expression by 2.3-fold compared to free RSV administration. This coordinated action destabilizes the Wnt/β-catenin-TCF4 transcriptional complex, reducing MYC and cyclin D1 expression critical for tumor proliferation [142, 143]. The hydrogel matrix enhances RSV's bioavailability through sustained local release, maintaining therapeutic concentrations for 72 h post-administration compared to 8 h for oral delivery [144]. Crucially, this delivery system synergizes with conventional chemotherapy by simultaneously inhibiting STAT3 and Akt pathways, as demonstrated in combination studies with 5-FU where apoptosis rates increased from 22 to 67% in HT-29 cell lines [145].

Glioblastoma treatment benefits from ROS-responsive hydrogels that exploit tumor microenvironment characteristics. These thioketal-based systems release 89% of encapsulated RSV within 4 h upon encountering glioblastoma-associated ROS levels (2.5-fold higher than normal brain tissue) [47, 48]. When co-administered with temozolomide (TMZ), the formulation demonstrates blood–brain barrier penetration efficiency 3.1 times greater than free TMZ by inhibiting ABCB1/ABCG2 efflux transporters [49]. The combinatorial approach reduces MGMT expression by 41% through RSV-mediated epigenetic modulation while enhancing TMZ's alkylating activity [50]. In orthotopic U87MG models, this dual delivery system decreases tumor volume by 78% compared to 52% with TMZ monotherapy, with complete EMT reversal evidenced by 4.8-fold increase in E-cadherin expression [51, 52].

Recent advancements (2020–2024) highlight innovative hydrogel designs optimizing RSV delivery. pH/thermo-responsive chitosan-PNIPAM hydrogels achieve 91% encapsulation efficiency through host–guest interactions with β-cyclodextrin [146]. ROS-sensitive PEG-PPS hydrogels with MXene nanosheets enable NIR-triggered release, combining photothermal therapy with RSV's anti-angiogenic effects [147]. Clinical translation potential is evidenced in 4D-printed multiresponsive hydrogels that maintain > 80% RSV stability for 28 days at physiological conditions while responding to tumor-specific MMP-9 and hyaluronidase [17]. These platforms address RSV's pharmacokinetic limitations, reducing hepatic first-pass metabolism from 74 to 12% through localized delivery [135].

The emerging paradigm leverages hydrogel-mediated spatial control of RSV release with molecular pathway precision, demonstrating 3.2–5.6 fold improvements in therapeutic index across preclinical models compared to conventional administration [148]. Ongoing challenges remain in optimizing degradation kinetics for different tumor microenvironments and scaling up GMP-compliant manufacturing processes for clinical evaluation [149, 150] (Table 2).

**Table 2:** Comparative analysis of RSV-loaded hydrogel systems for cancer therapy

Hydrogel type	Mechanical properties	In vivo half-life/degradation	Targeting mechanism	Tumor models tested	Key therapeutic outcomes
Chitosan-HA hydrogels	Compressive modulus: ≈1.5 MPa Elongation: ≈200% (PVA/HA/TA)	> 72 h sustained release	pH-responsive (tumor acidity: pH 6.5–7.0)	Hepatocellular carcinoma	↑ Intratumoral RSV accumulation by 40%; ↓ Off-target toxicity
Thermo-sensitive (F127-based)	Storage modulus (G'): > 10 kPa	24 h structural integrity	Temperature-responsive (37 °C gelation)	Melanoma, HCC	90% tumor suppression; Synergy with AuNPs for photothermal therapy
ROS-sensitive (Thioketal)	Shear strength: ≈3 × gelatin methacryloyl	4 h burst release (89% at high ROS)	ROS-triggered (2.5 × normal brain ROS levels)	Glioblastoma (U87MG)	78% ↓ tumor volume; Enhanced TMZ penetration (3.1 × free drug)
PEG-PPS/MXene nanocomposite	Tunable via crosslink density	> 28 days stability (> 80% RSV)	NIR-responsive + MMP-9/hyaluronidase sensitivity	Colorectal cancer	Complete EMT reversal (↑ E-cadherin 4.8 ×); Metastasis suppression via Wnt/β-catenin inhibition
Alginate immunogels	Swelling ratio: pH-dependent (max at pH 7.4)	14-day sustained release (70% Ab)	CTLA-4 blockade + DC maturation	Breast cancer (4T1)	↑ Tumor-infiltrating T cells 4.5 ×; ↓ Recurrence by 60%
Gelatin hydrogels	Gel strength correlates with release kinetics	72 h therapeutic concentration	Passive tumor retention	Colorectal cancer (HT-29)	↓ β-catenin nuclear translocation 58%; ↑ Apoptosis to 67% with 5-FU
Self-healing chitosan-cellulose	100% self-healing in 72 h	Not specified	ECM-mimetic scaffold	Neural injury models	Enhanced stem cell differentiation; Oxygen metabolism modulation

## Challenges and future perspectives

### Translational hurdles

Hydrogels have emerged as pivotal biomaterials in regenerative medicine and drug delivery, yet their clinical translation faces critical challenges in biocompatibility, degradation control, and scalable manufacturing. A primary translational hurdle lies in ensuring the long-term safety of hydrogel degradation byproducts. Recent studies reveal that while hydrogels like alginate-silica nanoparticle composites exhibit minimal inflammatory responses in hepatic and splenic tissues post-implantation, slight increases in apoptotic cells suggest potential side effects from degradation products or mechanical stress during injection. Similarly, TM/Dap hydrogels for traumatic brain injury demonstrate excellent biocompatibility with preserved liver and kidney function markers, yet their degradation kinetics must be optimized to balance therapeutic efficacy with byproduct clearance [151]. The interplay between degradation rate and bioactivity is exemplified by decellularized bovine pericardium (dBP) hydrogels, where rapid degradation releases immunomodulatory matrikines and extracellular vesicles, enhancing regenerative potential despite structural instability. However, sterilization methods such as gamma irradiation or ethylene oxide treatment risk altering hydrogel rheological properties and bioactivity, as evidenced by autoclaving causing significant increases in storage modulus (G') and loss modulus (G'') in alginate hydrogels due to water loss and structural disruption [152]. Terminal sterilization remains particularly problematic for hydrogels with temperature-sensitive components (e.g., PNIPAM) or bioactive peptides, where ethanol disinfection may preserve function but lacks guaranteed sterility, while UVC irradiation degrades RGD domains [153]. This underscores the need for tailored sterilization protocols that maintain sterility without compromising functionality [78].

Scalability challenges stem from batch-to-batch variability in pore architecture and drug-loading efficiency during upscaling. IPN hydrogels highlight the sensitivity of synthesis to environmental factors like temperature and humidity, where minor deviations in crosslinker reactivity or monomer ratios lead to inconsistent mechanical strength and swelling behavior [154]. Natural hydrogels exhibit significant batch variations due to source material heterogeneity, while synthetic hydrogels require stringent control over reaction conditions to minimize property fluctuations [155, 156]. For instance, scaling up lithium-alginate/poly(acrylamide-co-stearate) hydrogels requires precise control over ion-exchange sequences to maintain pore uniformity and mechanical resilience, as Li + substitution enhances carboxylate binding but demands rigorous process standardization [157]. Emerging strategies to mitigate variability include 3D printing technologies, which enable precise control over pore size (360–400 μm) and drug distribution through layer-by-layer fabrication of alginate/gelatin hybrids. Stimuli-responsive systems further improve drug-loading reproducibility; temperature-sensitive PNIPAM copolymers achieve controlled doxorubicin release via LCST-triggered structural transitions, while UV-crosslinkable hydrogels allow spatiotemporal modulation of drug elution profiles [158].

Regulatory challenges, including FDA requirements for biologics and medical devices, significantly delay clinical deployment. Hydrogels are typically classified as Class III medical devices under EU MDR or FDA regulations due to long-term tissue contact, requiring extensive premarket clinical investigations [159]. The 510(k) pathway demands demonstration of substantial equivalence, yet natural polymer hydrogels face near-zero FDA approval rates primarily due to unresolved batch variability and lack of pharmaceutical-grade biopolymer supply chains [160]. Regulatory rejections frequently occur over insufficient clinical data; for example, 83% of high-risk medical device rejections by Brazil's ANVISA in 2017 cited inadequate safety/efficacy evidence, a common hurdle for hydrogel-based combination products. The FDA's mandatory 510(k) review for most hydrogel scaffolds extends timelines to 7–10 years, critically undermining economic viability [161, 162]. These hurdles necessitate early integration of GMP considerations, including validation of sterilization efficacy and analytical methods to quantify batch consistency throughout the product lifecycle.

Future advancements hinge on integrating real-time degradation monitoring and adaptive manufacturing. Upconversion nanoparticles (UCNPs) embedded in hydrogels enable non-invasive tracking of degradation kinetics through near-infrared fluorescence, correlating hydrogel mass loss with signal attenuation in vivo. Concurrently, machine learning approaches are being explored to predict batch variability by analyzing synthesis parameters, potentially standardizing production across scales. As the field progresses, harmonizing material innovation with regulatory frameworks for degradation byproduct safety and sterilization validation will be essential to bridge the gap between laboratory breakthroughs and clinical adoption.

### Innovative directions

The field of cancer therapeutics is witnessing transformative advancements through the integration of smart biomaterials and precision gene-editing tools. Among the most promising innovations are 4D-printed hydrogels, which enable spatiotemporal control over drug release by responding to external stimuli such as magnetic fields or enzymatic activity. These hydrogels, composed of shape-memory polymers, dynamically alter their structure upon exposure to specific triggers, allowing programmable release profiles of therapeutic agents like oncolytic viruses (e.g., respiratory syncytial virus, RSV). For instance, poly(acrylic acid)-based hydrogels with Fe₃O₄ nanoparticles exhibit magnetic responsiveness, achieving rapid shape recovery (215 MPa Young's modulus) under alternating magnetic fields to modulate RSV release kinetics [163, 164]. Similarly, enzyme-responsive hydrogels utilizing MMP-cleavable motifs or glucose oxidase systems enable tumor microenvironment (TME)-specific activation, as demonstrated in pH-sensitive DNA hydrogels that release insulin in a glucose-dependent manner [165]. However, the translational potential of these systems is constrained by unresolved engineering challenges in 4D-bioprinting. Maintaining rheological stability of bioinks—particularly viscosity below 50 mPa·s to ensure jetting fidelity—requires stringent control over polymer molecular weight, solute–solvent ratios, and nanoparticle concentration, as deviations can lead to non-uniform deposition and compromised structural integrity. Furthermore, nozzle clogging remains a critical limitation, especially with high solid-content (> 3 wt%) or nanoparticle-loaded inks where gravitational settling and fiber bridging obstruct material flow; this necessitates innovative mitigation strategies like Plan B nozzle designs (reducing clogging by 100% at 0.35 mm-26.68% fill rates), ultrasonic cell manipulation, or dual-nozzle systems to ensure continuous operation [166]. 4D-printed hydrogels, composed of shape-memory polymers like poly(acrylic acid)-Fe₃O₄ composites, dynamically alter their structure under alternating magnetic fields (215 MPa Young’s modulus) to achieve programmable drug release [167]. Precision in layer deposition for in vivo implants demands micron-scale accuracy (optimal layer thickness: 0.09 mm) to conform to complex anatomical contours, requiring advanced path-planning algorithms, real-time current-based nozzle monitoring, and temperature-controlled deposition (e.g., 325 °C for ZnO layers) to minimize thermal distortion and ensure dimensional fidelity [168]. These systems exploit TME-specific triggers (e.g., MMP-9, hyaluronidase) to activate on-demand RSV delivery, as demonstrated in post-resection glioblastoma models where pH/thermo-responsive chitosan-PNIPAM hydrogels achieved 91% encapsulation efficiency [169]. CRISPR-Cas9 co-delivery platforms, such as liposome-templated hydrogel nanoparticles, enable simultaneous RSV-mediated chemosensitization and PLK1 gene knockout, synergistically suppressing MYC-driven osteosarcoma through synthetic lethality [170]. Critically, the sustained release kinetics inherent to hydrogels necessitate stringent management of CRISPR off-target activity, which is further complicated by potential inconsistencies in bioink deposition accuracy during multi-material printing. Such systems not only enhance therapeutic precision but also mitigate off-target effects, representing a paradigm shift in controlled drug delivery [167], provided these engineering hurdles are systematically addressed.

Concurrently, CRISPR-Cas9 co-delivery platforms embedded within hydrogels are emerging as dual epigenetic modulation tools. By encapsulating both RSV and CRISPR ribonucleoproteins (RNPs) in stimuli-responsive matrices, these systems enable simultaneous viral oncolysis and targeted gene editing. For example, liposome-templated hydrogel nanoparticles (LHNPs) efficiently co-deliver Cas9/sgRNA complexes and RSV, achieving synergistic tumor suppression in glioblastoma models through PLK1 gene knockout and viral replication [171]. Nevertheless, the functional success of such platforms hinges on overcoming bioprinting-specific barriers: shear-thinning bioinks (non-Newtonian behavior is ideal for jetting) must retain stability during high-shear deposition (10–100 kHz), while clogging-prone formulations demand dynamic viscosity monitoring via high-speed imaging or capillary rheometry to prevent print failures. Critically, the sustained release kinetics inherent to hydrogels necessitate stringent management of CRISPR off-target activity. This is addressed through multiple strategies: (i) using high-fidelity Cas9 variants (e.g., HiFiCas9, evoCas9) with reduced non-specific DNA binding; (ii) optimizing sgRNA design via computational tools (e.g., CRISPR-P 2.0, CRISPR-GE) to minimize off-target sites; and (iii) employing RNP delivery (rather than plasmid DNA) to limit duration of nuclease activity, thereby mitigating off-target effects [172, 173]. Furthermore, the risks of genomic integration—a concern with viral vectors—are minimized in hydrogel systems due to the non-integrative nature of RNPs and transient Cas9 expression. Linear DNA designs (e.g., CELiDs) and self-inactivating lentiviral backbones further reduce integration frequencies, lowering risks of insertional mutagenesis [174, 175].

Immunogenicity remains a pivotal challenge, as pre-existing anti-Cas9 antibodies are reported in 5–95% of the population [176]. Hydrogel encapsulation attenuates this risk by shielding Cas9/sgRNA complexes from rapid immune recognition. Additionally, strategies like PEGylation of delivery systems and selection of less immunogenic Cas orthologs (e.g., SaCas9) enhance biocompatibility [177, 178]. This approach addresses oncogenic miRNA dysregulation—a hallmark of stromal-rich tumors—by combining viral-induced immunogenic cell death with CRISPR-mediated epigenetic silencing. However, rigorous in vivo immunogenicity profiling and long-term safety studies of CRISPR-hydrogel platforms remain essential, particularly concerning unintended immune activation or cellular toxicity in non-target tissues [179].Such systems not only enhance therapeutic precision but also mitigate off-target effects, representing a paradigm shift in controlled drug delivery [180].

Translating these innovations into clinical practice necessitates prioritizing phase I/II trials for recalcitrant cancers with dense stromal microenvironments, such as pancreatic adenocarcinoma and glioblastoma. The fibrotic TME in these malignancies imposes dual barriers: limited drug penetration and immunosuppressive stromal signaling. Preclinical models demonstrate that RSV-hydrogel hybrids, when combined with stroma-modulating agents (e.g., PDGFRα/β inhibitors), enhance intratumoral drug accumulation by threefold while reducing interstitial fluid pressure [181]. Notably, 4D-printed chitosan-CuNP hydrogels have shown efficacy in post-resection glioblastoma models, crossing the blood-tumor barrier under NIR irradiation to reduce relapse rates by 60% [127]. Future trials should explore sequential therapy regimens, where initial stromal disruption via photodynamic or Hedgehog pathway inhibition primes tumors for subsequent RSV-CRISPR hydrogel therapy, capitalizing on transient vascular normalization windows [182]. Challenges remain in optimizing hydrogel degradation rates and CRISPR payload stability, but emerging bioink technologies and in vivo CRISPR screening tools offer pathways to clinical translation [183].

## Conclusion

The integration of resveratrol with hydrogel-based delivery systems offers a promising strategy to overcome pharmacokinetic limitations of conventional cancer therapies. Hydrogels enhance RSV’s stability, prolong its half-life, and enable tumor-specific release through stimuli-responsive mechanisms, thereby amplifying its anticancer efficacy in preclinical models. By synergizing with chemo-photothermal agents, immunomodulators, and gene-editing tools, RSV-hydrogel platforms demonstrate versatility in suppressing metastasis and reversing chemoresistance. Notably, successes in colorectal cancer and glioblastoma models highlight improved therapeutic indices and reduced off-target toxicity. Nevertheless, the translation of these platforms faces substantial hurdles that temper clinical optimism. Batch variability, unpredictable degradation kinetics, and regulatory complexities necessitate interdisciplinary collaboration to bridge the well-documented gap between laboratory innovation and clinical application. Only ~ 5% of biomedical discoveries achieve clinical translation, underscoring the need for cautious interpretation of preclinical data.

Future innovations—such as AI-driven optimization of hydrogel degradation kinetics and 4D-printed implants—must concurrently address manufacturing scalability and biocompatibility validation. Machine learning algorithms trained on compositional datasets (e.g., crosslinker density, polymer ratios) could predict drug release profiles for personalized implants. However, AI-driven biomaterial design introduces ethical challenges, including algorithmic bias in data curation and accountability for unintended clinical consequences. Moreover, GMP-compliant manufacturing and long-term biocompatibility assessments remain critical barriers to clinical adoption, as few hydrogel systems achieve FDA approval due to heterogeneous inflammatory responses and biodegradation mismatches.

While RSV-hydrogel synergies exemplify the convergence of biomaterial engineering and phytochemical pharmacology, their transformative potential in oncology depends on resolving translational bottlenecks. Collectively, these systems represent a conceptual blueprint rather than an immediate solution, requiring rigorous validation of combinatorial strategies (e.g., stromal modulation) in complex human pathophysiology.

## Data Availability

No datasets were generated or analysed during the current study.
